# Reduction of Endogenous Melatonin Accelerates Cognitive Decline in Mice in a Simulated Occupational Formaldehyde Exposure Environment

**DOI:** 10.3390/ijerph13030258

**Published:** 2016-02-26

**Authors:** Yufei Mei, Chunli Duan, Xiaoxiao Li, Yun Zhao, Fenghua Cao, Shuai Shang, Shumao Ding, Xiangpei Yue, Ge Gao, Hui Yang, Luxi Shen, Xueyan Feng, Jianping Jia, Zhiqian Tong, Xu Yang

**Affiliations:** 1 Section of Environmental Biomedicine, Hubei Key Laboratory of Genetic Regulation and Integrative Biology, College of Life Sciences, Central China Normal University, Wuhan 430079, China; meiyufei1990@foxmail.com (Y.M.); lixiaoxiao201409@sina.com (X.L.); 18735501894@163.com (Y.Z.); cfh723@163.com (F.C.); sddy06wendy@sohu.com (S.S.); dingsm@mail.ccnu.edu.cn (S.D.); 2 Alzheimer’s Disease Center, Beijing Institute for Brain Disorders, Capital Medical University, Beijing 100069, China; xiangpei2015@sina.com (X.Y); imaginary-diva@163.com (L.S.); feng402@139.com (X.F.); jjp@ccmu.edu.cn (J.J.); 3 Department of Neuobiology, Capital Medical University, Beijing 100069, China; cldduan@ccmu.edu.cn (C.D.); gaog@ccmu.edu.cn (G.G.); huiyang@ccmu.edu.cn (H.Y.)

**Keywords:** formaldehyde (FA), melatonin (MT), oxidative stress, spatial memory, reactive oxygen species, l-glutathione

## Abstract

Individuals afflicted with occupational formaldehyde (FA) exposure often suffer from abnormal behaviors such as aggression, depression, anxiety, sleep disorders, and in particular, cognitive impairments. Coincidentally, clinical patients with melatonin (MT) deficiency also complain of cognitive problems associated with the above mental disorders. Whether and how FA affects endogenous MT metabolism and induces cognitive decline need to be elucidated. To mimic occupational FA exposure environment, 16 healthy adult male mice were exposed to gaseous FA (3 mg/m^3^) for 7 consecutive days. Results showed that FA exposure impaired spatial memory associated with hippocampal neuronal death. Biochemical analysis revealed that FA exposure elicited an intensive oxidative stress by reducing systemic glutathione levels, in particular, decreasing brain MT concentrations. Inversely, intraperitoneal injection of MT markedly attenuated FA-induced hippocampal neuronal death, restored brain MT levels, and reversed memory decline. At tissue levels, injection of FA into the hippocampus distinctly reduced brain MT concentrations. Furthermore, at cellular and molecular levels, we found that FA directly inactivated MT *in vitro* and *in vivo*. These findings suggest that MT supplementation contributes to the rescue of cognitive decline, and may alleviate mental disorders in the occupational FA-exposed human populations.

## 1. Introduction

Although formaldehyde (FA) is generally considered an environmental pollutant [1] and neurotoxic substance [2], it is an indispensable raw material for hospital preservation, industrial activities, and agriculture disinfectant [3]. Hence, a large number of workers are exposed to FA [4]. Epidemiological studies have shown that work-related exposure to FA results in headaches, anxiety, fatigue, sleep disorders, and in particular, cognitive disorders [5,6]. Accordingly, the results of animal experiments reveal that gaseous FA exposure induces abnormal behaviors, such as: aggression, depression, a decline in locomotor activity, and spatial memory deficits [7,8,9]. Surprisingly, studies have indicated that healthy rats that have been exposed to abnormally high concentrations of gaseous FA have normal levels of FA in the brain [10,11,12]. Therefore, which endogenous factor is the molecular target of FA remains largely unknown.

Endogenous melatonin (MT), *N*-acetyl-5-methoxytryptamine, is a hormone present in mammalian brains, including humans [13,14,15], which is involved in the entrainment (synchronization) of circadian rhythms of various physiological functions including sleep patterning, blood pressure regulation, moodiness [16,17], and memory [18]. As well as occupational FA-exposed workers, those with mental abnormalities, clinical patients with mild cognitive impairments, and Alzheimer’s disease (AD), also exhibit abnormal behaviors such as aggression or depression, anxiety, insomnia, and cognitive decline [19,20,21]. Notably, a marked elevation in FA levels [22,23] associated with a reduction in MT is observed in the serum and postmortem cerebrospinal fluid of patients suffering from AD [24,25,26]. Previous studies suggest that application of MT can reverse cognitive decline in FA-exposed animal models [27,28], and mental illness in human [29,30,31]. These data strongly suggest that endogenous MT deficiency is a possible reason for occupational FA exposure-related cognitive impairments and mental disorders.

In the present study, we found that under a simulated occupational FA exposure environment, mice exposed to FA displayed spatial memory decline associated with MT reduction in the brains. Furthermore, both *in vitro* and *in vivo* experimental results indicated that FA can directly inactivate MT. Therefore, supplementation of MT (a powerful brain antioxidant), can reduce the effects of FA-induced brain oxidative stress, and possibly reverse cognitive impairments. Finally, possible roles of deficiencies in brain derived MT in mental disorders are also discussed.

## 2. Methods

### 2.1. Animals

All specified pathogen-free adult male Bal b/c mice (6 weeks old, 18–20 g) were provided by the Experimental Animal Center of Hubei Province (Wuhan, China) and housed in standard conditions (12 h light-dark cycle with lights on at 8:00 am, off at 20:00 pm, 70%–80% humidity, 25 ± 1 °C) and food and water were provided *ad libitum*. All animal experiments were conducted in accordance with National Institutes of Health Guide for the Care and Use of Laboratory Animals, and were approved by the Office of Scientific Research Management of Central China Normal University (1 March 2012 CCNU-IACUC-2012-011).

### 2.2. Reagents and Kits

Formaldehyde and MT were purchased from Sigma—Aldrich (St Louis, MO, USA). All other chemicals were analytically pure. The l-glutathione (GSH) kit was purchased from Nanjing Jiancheng (Nanjing, China). The cell counting kit-8 (CCK-8) was purchased from Biosharp Company (Nanjing, China).

### 2.3. Experimental Protocol

Thirty-two mice were randomly divided into four groups (*n* = 8, per group): (1) control group (Con), intraperitoneal injected (i.p.) with saline; (2) gaseous FA exposure group, exposed to FA (3 mg/m^3^); (3) the MT group, injected with MT (10 mg/kg; MT); (4) the FA exposure combined with MT injection group (FA + MT). MT was i.p. injected at 8:00 pm for 7 consecutive days. Two groups of mice were put into a gaseous FA exposure chamber (Wuhan, China) and exposed to 3 mg/m^3^ of gaseous FA from 8:00 am to 16:00 pm, 8 h a day for 7 consecutive days. During gaseous FA exposure, mice were not allowed to drink or eat, in order to avoid gaseous FA dissolving in water. The FA exposure chamber was kept under normal conditions (1.00 ± 0.01 L/min gas flux, 40%–50% humidity, 25 ± 1 °C) and the average concentrations of gaseous FA were maintained at 3.04 ± 0.13 mg/m^3^, a level that has been found to be neurotoxic (9), Measurements were calculated using an interscan 4160 digital electrochemical analyzer (Chatsworth, CA, USA) every 2 h.

### 2.4. Morris Water Maze Test

Memory-related behaviors of mice were analyzed using the Morris water maze. Following exposure to gaseous FA, mice were transferred to the Morris water platform, and spatial training and memory retrieval experiments were conducted as previously described [22,32]. Spatial training in the Morris water maze was performed 3 h later after 8-h FA exposure from day 1 to 7, and the probe test was carried out on day 8.

### 2.5. Preparation of Brain Tissue Homogenates and Histological Analysis 

After the Morris water maze test, all mice were anesthetized with pentobarbital sodium (10 mg/kg, i.p.) and sacrificed by cervical dislocation. Brains were immediately removed with medical scissors, rinsed in 10 mL/g of ice-cold phosphate buffer (PBS, 0.1 mM) and separated into two parts. The first halves were immersed in 10% paraformaldehyde for histological analysis. After two days, these paraformaldehyde fixed brain tissues were stained with hematoxylin and eosin (H&E) and analyzed as previously described while the other halves were homogenized for biochemical analysis. The homogenates were centrifuged at 12,000× *g* for 10 min at 4 °C and frozen at −70 °C until further use [33]. 

### 2.6. Determination of FA by UV-HPLC

Brain tissue homogenates were thawed at room temperature (25 °C) on ice. An aliquot of 0.5 mL sample was added with 0.5 mL 10% trichloroacetic acid (*v*/*v*, in water) in a 2 mL centrifuge tube, and then vortexed for 5 min. After centrifugation (12,000× *g*), 4°C, 30 min), an aliquot of 0.4 mL of supernatant was pipetted into a 2 mL vial, and 0.1 mL of 2,4-dinitropheylhydrazine (1 mg/mL ,in acetonitrile) and 100% acetonitrile were added. The vials were capped tightly and vortexed for 30 s, and bathed in water for 30 min at 60 °C and centrifuged at 12,000× *g*, for 10 min at 4 °C. Finally, the supernatant aqueous phase was extracted for high performance liquid chromatography with ultraviolet spectroscopy analysis [34].

### 2.7. Detection of MT Levels Using ELISA Kit

Supernatants of brain homogenates were used to detect MT concentrations according to the manufacturer’s instructions (BYE30198, Bangyi Biotech, Shanghai, China). 10 μL samples with 40 μL sample dilution were added into wells, and incubated for 30 min at 37 °C. Then washing buffer was added to each well for 30 s, then drained, repeated for 5 times, and dried by pat. 50 μL HRP-conjugated reagent were added to each well except blank well, and incubated for 30 min at 37 °C. The washing buffer was added to each well for 30 s, and then drained, repeated 5 times, and dried by pat. 50 μL chromogen solution A and 50 μL chromogen solution B were added to each well, and evaded the light preservation for 15 min at 37 °C. Then 50 μL stop solution was added into each well. The blank well was taken as zero. The absorbance at 450 nm of the plate was read after adding stop solution. The concentration of MT in the samples was determined by the standard curve.

### 2.8. Intracerebroventricular Injection of Reagents in Mice

Cannulae were stereologically inserted into the brains of six week old mice at 0.5 mm anterior/posterior to bregma, 1.0 mm medial/lateral to the midsagittal suture, and 2.0 mm dorsal/ventral from the brain surface. A sterile head was pulled out before each injection, and a new one was inserted cannulae for avoiding contamination after each injection. An i.c.v. injection of either normal saline, FA (100 μM, 2 μL was injected for 10 min), MT (100 μM, 2 μL, for 10 min) or FA with MT (100 μM, 2 μL, for 10 min) were administered [22]. Three hours after injections, brains were collected for the detection of MT and FA levels.

### 2.9. Reactive Oxygen Species (ROS) Assay

Reactive oxygen species (ROS) was measured using oxidation-sensitive fluorescent DCFH-DA, which is a non-fluorescent compound that is freely taken up by cells and hydrolyzed by esterases to 2′, 7′—dichlorodihydrofluorescein (DCFH). DCFH is then oxidized to the fluorescent dichlorofluorescein (DCF) in the presence of peroxides, thereby indicating the level of intracellular ROS. Briefly, the supernatant was diluted ten times with PBS (pH = 7.4), then 100 μL was removed to a 96-well microplate, and 100 μL of 10 μmol/L DCFH-DA was added. The reaction mixture was kept in the dark for 30 min at 37 °C, and then the fluorescence intensity was measured at an excitation wavelength of 488 nm and an emission wavelength of 525 nm by a fluorescence reader (FLx800, BioTek Instruments, Vinooski, VT, USA).

### 2.10. l-glutathione (GSH) Assay

The GSH content and all protein concentrations were measured using the Lowry assay method and were determined as previously described [35].Glutathione (GSH) can react with DTNB in the dark and produces 2-nitro-5-thiobenzoic acid (TNB). In case of disturbance by thiols in proteins, 10% tichloroacetic acid was used to delimitate these proteins. Afterwards, the pH was adjusted to 7.5 to yield the color-change reaction with DTNB (60 μg/mL), 50 μL supernatant was transferred into the 96-well microplate, 150 μL of DTNB added, and then incubated in the dark at room temperature for 5 min. Experimental and standard samples were analyzed using a microplate reader at a wavelength of 412 nm. Based on the standard curve, the calculation was GSH (nmol/L) = (OD_412_–0.0524)/0.0049, *R*^2^ = 0.994.

### 2.11. Cell Culture and Cell Viability Assay

The mouse neuroblastoma (N2a) cell line was purchased from the Center for Cell Collection (Wuhan, China). The N2a cells were cultured in Dulbecco’s Modified Eagle’s Medium (DMEM) medium containing 10% (v/v) fetal bovine serum, and passaged every 2 days and maintained in a humidified atmosphere of 95% air, 5% CO_2_ at 37 °C. The N2a cells were seeded into a 96-well plate at a density of 1 × 10^5^ cells /mL (200 μL) per well. Cells were allowed to adhere and after 24 h, the medium was replaced with fresh DMEM containing FA at 0, 50, 150, 250, and 450 μM, or MT at 0, 10, 50, 150, and 250 μM. Cell viability was assessed using a CCK8-kit according to the manufacturer’s instructions.

### 2.12. Chemical Reaction between FA and MT

The MTsolutions were prepared by dissolving powder in 10 mM PBS pH 8.0 and were stored at 4 °C until further use. The compounds FA (0.01 mM, 1 mL), MT (0.01 mM, 1 mL), and a mixture of these two compounds at 0.01 mM were dissolved in PBS at 37 °C for 2 hand all solutions were used for detecting FA levels.

### 2.13. Statistical Analysis

Graphs were generated using GraphPad Prism version 5.01 (GraphPad Software Inc., San Diego, CA, USA). Statistical analyses were performed using SPSS software version 21.0 (SPSS Inc., Chicago, IL, USA). In the Morris water maze experiment, Fisher’s LSD was used for post hoc comparisons, and the difference between different treatments groups within each day were analyzed using a one-way ANOVA. For other experiments, statistical significance was determined using a one-way ANOVA followed by Tukey’sposhoc test. Data are represented as mean ± standard error over three independent experiments. *p* < 0.05 was considered as significant value.

## 3. Results

### 3.1. Treatment with MT Reverses Gaseous FA Exposure-Induced Memory Deficits

**Figure 1:**
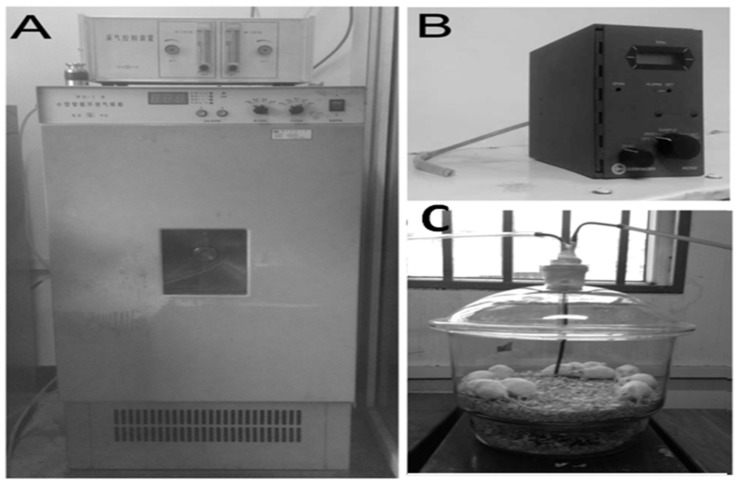
Mice inhale gaseous FA under a simulated occupational FA exposure environment. (**A**) An intelligentized environmental chamber for generating gaseous FA at 3.0 mg/m^3^; (**B**) An interscan 4160 digital electrochemical analyzer for detecting gaseous FA concentrations; (**C**) A modified 8.4 L of glass inhalation chamber for mimicking occupational FA exposure microenvironments.

To mimic the occupational FA exposure environment, we used a typical intelligentized environmental chamber for generating gaseous FA at 3.0 mg/m^3^ (Figure 1A), and gaseous FA concentrations were detected by an interscan 4160 digital electrochemical analyzer (Figure 1B). Four groups of healthy adult male mice were put into a modified 8.4 L glass inhalation chamber and exposed to gaseous FA or air (Figure 1C).

**Figure 2:**
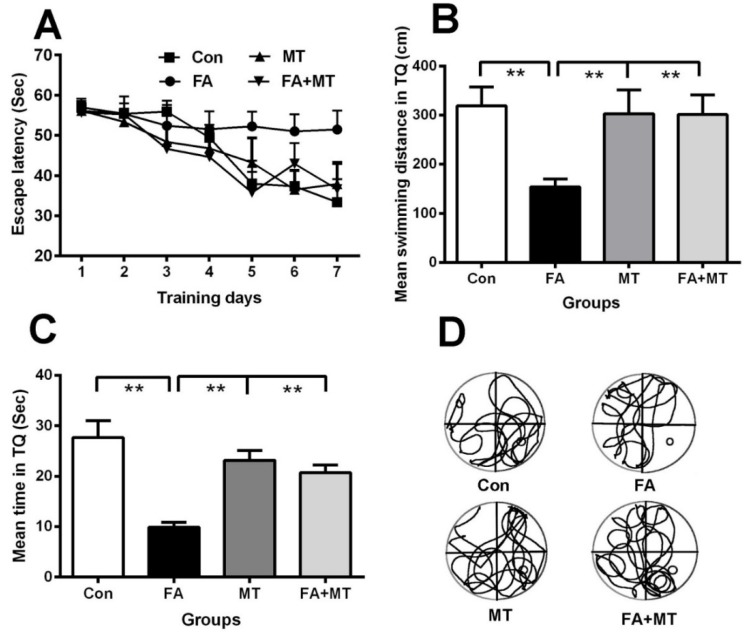
Injection of MT reverses gaseous FA exposure-induced spatial memory decline in mice. (**A**) Different effects of different reagents on the escape latency among control, FA exposure, MT injection alone, and FA exposure with MT (FA+MT) treated groups after 7days of spatial training (*n* = 8 for each group); (**B**) Different mean swimming distances in target quadrant (TQ) in the above four groups of mice on day 8 (*n* = 8 for each group); (**C**) Different mean times staying in TQ in these mice on day 8 (*n* = 8 for each group); (**D**) Swimming tracks of the four groups on day 8 (*n* = 8 for each group). ** *p* < 0.01.

To test our hypothesis that MT attenuates FA exposure-induced memory decline, we examined the memory behaviors of mice exposed to gaseous FA for 7 consecutive daysusing the Morris water maze. The two-way repeated measures ANOVA for the escape latency parameter revealed a main effect of day (*F*_5, 75_ = 56.525, *p* < 0.001), group (*F*_3, 28_ = 5.438, *p* < 0.01), and a day × group interaction (*F*_15, 75_ = 3.845, *p* < 0.05). Post hoc Dunnett tests showed a significant difference in swimming latency between the FA-exposed groups and control mice on acquisition day 5 (*F*_3, 28_ = 4.632, *p* < 0.001), day 6 (*F*_3, 28_ = 5.522, *p* < 0.001), and day 7 (*F*_3, 28_ = 9.971, *p* < 0.001) (Figure 2A). These data indicate that the mice exposed to gaseous FA have a lower spatial learning ability than the control mice, and MT injection reverses impaired learning abilities associated with exposure to gaseous FA. 

On day 8, results from the probe test indicated that the mice exposed to gaseous FA displayed a poorer memory retrieval ability, *i.e.*, a shorter swimming distance and less time spent in the target quadrant compared with the control mice (Figure 2B–D). Notably, an i.p. injection of MT markedly improved memory performance of mice exposed to gaseous FA (*n* = 8, *p*< 0.01; one-way ANOVAs) (Figure 2B–D). These data demonstrate that MT treatment reverses impaired memory retrieval ability associated with exposure to gaseous FA.

### 3.2. Treatment with MT Attenuates Hippocampal Neuronal Damage Induced by Exposure to FA

**Figure 3:**
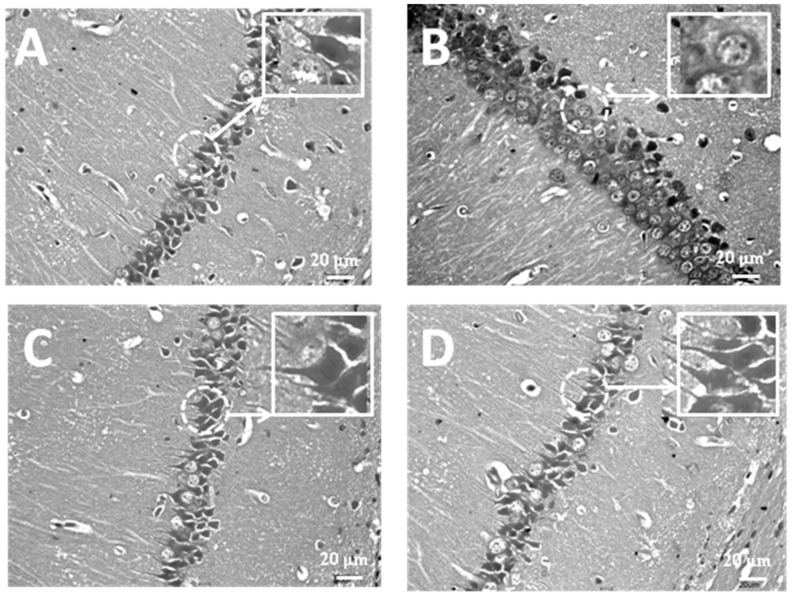
Histological observation shows gaseous FA exposure-induced hippocampal neurons death. (**A**) Control (Con); (**B**) FA exposure (FA); (**C**) MT injection (MT); (**D**) FA exposure with MT injection (FA + MT). Magnification: × 40. Bar: 20 μm.

Since the hippocampus is essential for spatial memory formation in rodents, we investigated whether gaseous FA exposure induces morphological alterations in hippocampal pyramidal neurons. The results of H&E staining showed that there were many robust neurons with normal neuronal cell bodies and long axons in pyramidal cell layer regions in the CA1 of control group mice (Figure 3A). However, in mice exposed to gaseous FA, the pyramidal cells underwent pyknosis, apoptosis, and in particular, the pyramidal cell layer was loosely packed, and karyopyknosis fragments were detected throughout the hippocampal CA1 region (Figure 3B). Inversely, these neuronal pathological features were clearly alleviated in pyramidal cell layer regions in the CA1 when exposure to FA was combined with MT treatment (FA + MT), compared with FA-exposed mice. In MT alone injected mice, there were less cellular pyknosis and apoptotic bodies in the CA1 region of hippocampi than in mice exposed to FA (Figure 3C,D). These results indicate that supplementation of MT improves the survival of hippocampal neurons. 

### 3.3. Application of MT Restores Brain MT Concentrations in Gaseous FA-Exposed Mice

**Figure 4:**
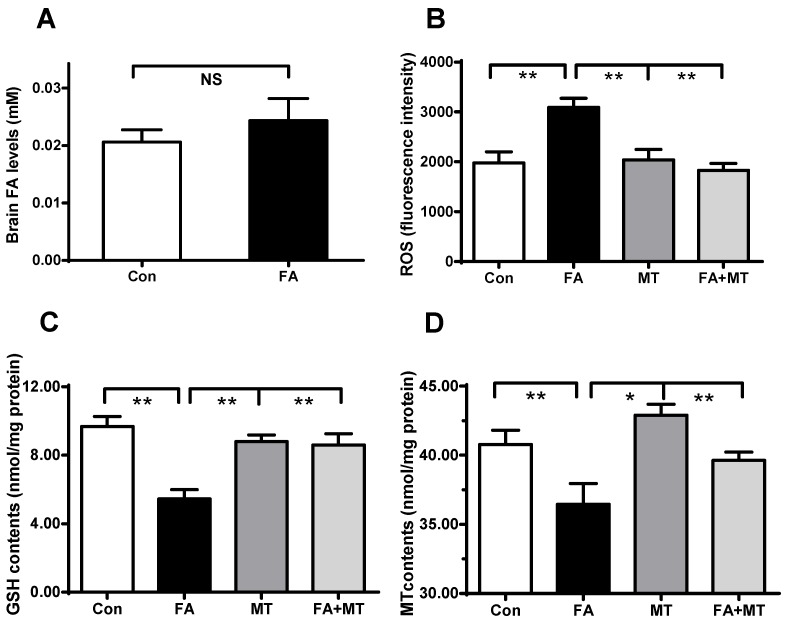
Changes in brain FA, ROS, GSH, and MT levels in control, gaseous FA-exposure, and FA exposure with MT (FA+MT) treatment group mice. (**A**) Brain FA levels in the control and gaseous FA-exposed group (*n* = 8 for each group); (**B**) The fluorescence intensity of ROS (*n* = 8 for each group); (**C**) Brain GSH levels in the four groups(*n* = 8 for each group); (**D**) Brain MT concentrations in the four groups (*n* = 8 for each group). * *p* < 0.05; ** *p* < 0.01.

To explore why MT supplementation can restore memory deficits in gaseous FA-exposed mice, we examined the changes in the concentrations of FA, ROS, GSH, and MT in the brains of the four experimental groups. First, we examined whether gaseous FA exposure induced an elevation in FA levels in the brains of mice. As has been previously reported [12], 7 days of gaseous FA exposure did not elicit a statistically significantly change in brain FA concentrations, although there was a slight increase compared with controls (Figure 4A). Since FA-induced oxidative stress is considered to contribute to memory deficits, we measured the levels of ROS, a typical marker of oxidative stress in mice. The results showed that gaseous FA exposure induced a distinct increase in brain ROS levels, and MT treatment reversed this increase (Figure 4B).

Next, we detected the levels of brain GSH, as oxidative stress is often accompanied with a decrease in systemic GSH concentrations. The results showed that gaseous FA exposure led to a 50% decrease in GSH levels, but MT treatment restored brain GSH levels (Figure 4C). Furthermore, we investigated whether gaseous FA exposure affects the metabolism of endogenous MT, a powerful antioxidant in the brain. Remarkably, brain MT levels were clearly decreased in gaseous FA-exposed mice, and MT supplementation restored brain MT levels (Figure 4D). These data suggest that FA exposure not only reduces systemic antioxidant GSH levels, but also depletes brain antioxidant MT concentrations.

### 3.4. An i.c.v. Injection of Liquid FA Induces Endogenous MT Reduction in the Brain 

**Figure 5:**
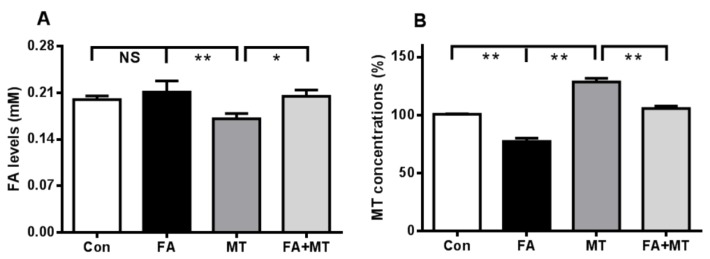
Changes in brain FA and MT after intracerebroventricular injection of liquid FA or FA combined with MT treatment for 3 h. (**A**) A decline in brain FA levels after 0.1 mM MT injection (*n* = 8 for each group); (**B**) A decline in brain MT levels after 0.1 mM FA injection, and restoration of brain MT levels after FA combined with MT treatment (*n* = 8 for each group). * *p* < 0.05; ** *p* < 0.01.

To explore whether FA is the direct factor for brain MT decline, we injected excess FA (0.1 mM, over blood FA level: 0.08 mM, i.c.v. [36]) into the brains of mice, and the levels of both FA and MT were detected in brain tissue 3 h after injection. Similar to the above results regarding gaseous FA exposure (Figure 4A), there was no a statistically significant difference in brain FA concentration between the liquid FA-injected group and the control group (Figure 5A). However, excess FA elicited a significant decrease in brain MT levels (Figure 5B), but supplementation of MT (i.p. before FA injection 30 min) restored brain MT contents in the FA-injected mice (Figure 5B**)**. Notably, MT treatment also induced a decline in brain FA levels (Figure 5A). These data suggest that FA may directly induce a decline in brain MT levels.

### 3.5. Liquid FA Directly Inactivates MT in Cultured Cells and in Vitro

**Figure 6:**
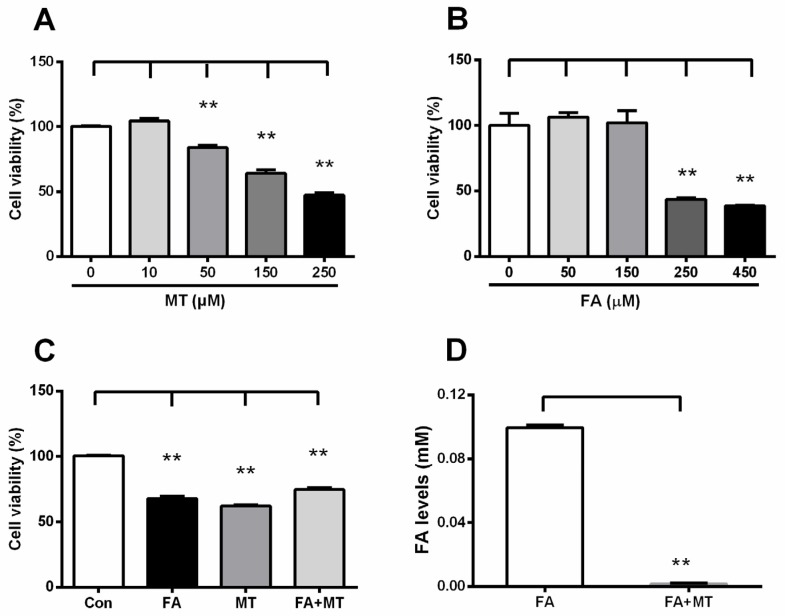
Liquid FA inactivates MT in cultured N2a cell line and *in vitro*. (**A**,**B**) A dose-dependent decline in cell viability after MT or FA alone treatment for 24 h (*n* = 6 for each group); (**C**) Changes in cell viability after 0.25 mM FA, 0.25 mM MT, and FA combined with MT (*n* = 6 for each group); (**D**) A chemical reaction between 0.01 mM FA and 0.01 mM MT in PBS at 37 °C (*n* = 6 for each group). ** *p* < 0.01.

We further explored the relationship between FA and MT at the cellular and molecular level. The results showed that supplementation of MT or FA alone induced a dose-dependent decrease in N2a cell viability after incubation with each reagent for 24 h (Figure 6A,B). However, application of MT prior to liquid FA incubation reversed FA-induced N2a cell death (Figure 6C). This result suggests that FA may directly interact with MT. In support of this proposed interaction, we found that incubation of these two compounds in PBS solution at 37 °C resulted in a spontaneous chemical reaction between liquid FA and MT *in vitro*(Figure 6D). In summary, these data indicate that FA can inactivate MT *in vitro* and *in vivo*. 

## 4. Discussion

**Figure 7:**
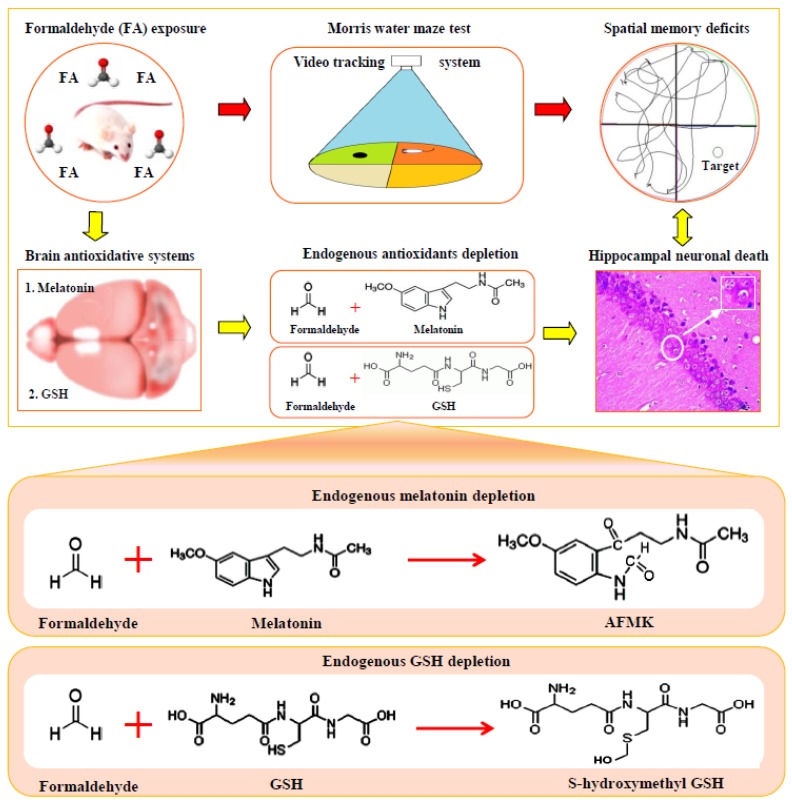
Occupational formaldehyde exposure induces spatial memory deficits by depleting antioxidants GSH and MT in the brains of mice.

In the present study, we focused on a critical question, namely whether cognitive decline in occupational FA exposed populations is directly related to exposure to FA. Using a gaseous FA exposure mouse model, we found that a reduction in both systemic antioxidant GSH and brain antioxidant MT contributes to cognitive impairments (Figure 7). Consistent with expectations, supplementation of MT can restore cognition in these gaseous FA-exposed mice. Meanwhile, we speculate that reduction of brain MT is most likely a pathological factor for mental disorders, such as aggressive behavior, depression, anxiety, fatigue, and insomnia that are found in occupational FA-exposed workers. 

Accumulating evidence shows that oxidative stress in the brain is involved in cognitive decline in rodents and humans [28,37]. For example, ROS, a classic marker of oxidative stress [38], which is considered to mediate neuronal degeneration and death [39], is observed to be abnormally elevated in liquid FA-incubated cells [40,41], gaseous FA-exposed mice [9,42], liquid FA-injected rats [43], and most importantly, in the occupational FA-exposed workers [44]. Inversely, GSH, a systemic antioxidant [45], is markedly decreased in animal models and humans after FA exposure [9,46]. This is because endogenous GSH spontaneously has a chemical reaction with FA *in vitro* and *in vivo* [47]. Notably, brain MT, a powerful antioxidant in brains [48,49], is observed to be decreased in gaseous FA-exposed mice in this study, as well as AD patients [24]. Unsurprisingly, excess hippocampal FA has been found in autopsied samples from AD patients [32,50]. Most importantly, both gaseous FA exposure and liquid FA injection impairs hippocampal structure [28,36], which is essential for spatial memory formation [51]. In this study, MT supplementation can reduce brain oxidative stress, attenuate hippocampal structural damage, and also restore gaseous FA exposure-induced cognitive decline in mice. The latter result is consistent with an observation in a previous report where a liquid FA-injected rat model was used [27,28]. These data confirm that brain MT reduction contributes to occupational FA exposure-related cognitive decline.

A possible explanation for mental disorders in the occupational FA-exposed populations is that FA-inactivated MT leads to a decline in brain MT levels. Previous study suggests that there is a final product formation (N1-acetyl-N2-formyl-5-methoxykynuramine, AFMK) between MT and free radicals such as HO· [52]. FA, a strong oxidizing agent, most likely attacks the same indole ring of MT as well as HO· (Figure 7), which requires further investigation. Brain MT, a hormone secreted by the pineal gland [13], has been previously found to be related to regulating mood in mammals [53,54]. Decreased levels of MT accompanied by memory decline were observed in aged and neurodegenerative diseased individuals [55,56]. Interestingly, in older humans, a decline in MT levels is often associated with sleep disturbances [55]. Clinically, patients with MT deficiency exhibit mental disorders, such as aggressive behaviors, depression, anxiety, sleep disorders, and in particular, cognitive impairments [53,57]. Results from the present study regarding FA exposure and lowered levels of brain MT may explain why occupational FA exposed-people often suffer from anxiety, fatigue, insomnia, and memory disturbances [5,6]. In animal models, low doses of gaseous FA exposure indeed induces anxiety-like and depression-like behavior [21], aggression [8], and memory dificents [21,22], but both high doses of gaseous FA exposure and liquid FA injection elicit depression [7] and memory decline [9,23]. In this study, we also found that the gaseous FA-exposed mice displayed abnormal behaviors, such as less active exploration, huddling motionless, and memory deficits. However, MT supplementation reversed these abnormal mental symptoms [58]. Similarly, the positive therapeutic effects of MT application have also been observed in clinical AD patients with mental disorders [35,59].

## 5. Conclusions

In conclusion, occupational FA exposure directly induces brain MT reduction, which is most likely related with cognitive decline and mental disorders. Therefore, MT supplementation is a potential strategy for preventing gaseous FA-induced neurotoxicity in occupational FA-exposed populations.
